# Supplementary material for: Consumer and provider perceptions of the specialist unit model of care: A qualitative study
Source: PLoS One. 2024 Feb 12;19(2):e0293025. doi: 10.1371/journal.pone.0293025 (PMC10861032; doi:10.1371/journal.pone.0293025)
Supplement: S2 File — (PDF) [file pone.0293025.s002.pdf]

Supplementary File 2

List of high impact conditions prevalent in the health facility catchment.

- Cardiac arrest, chest pain, acute myocardial infarction, congestive heart failure.
- Fractures, knee replacement, hip replacement, joint replacement, abnormal gait, bone disease, osteoporosis.
- Abdominal pain, pelvic pain, gastrointestinal pain.
- Pneumonia, asthma, chronic obstructive pulmonary disease.
- Postnatal depression.
- Dialysis (haemodialysis etc.), kidney disease, end stage kidney disease.
