# Supplementary material for: Consumer and provider perceptions of the specialist unit model of care: A qualitative study
Source: PLoS One. 2024 Feb 12;19(2):e0293025. doi: 10.1371/journal.pone.0293025 (PMC10861032; doi:10.1371/journal.pone.0293025)
Supplement: Attachment — Submitted filename: PONE-D-23-12601_Review_22.05.23_.pdf [file pone.0293025.s003.pdf]

Review for manuscript: Consumer and provider perceptions of the specialist unit model of care: a qualitative study

PLOS ONE

Thank you for the opportunity to review this manuscript. I enjoyed reading the article and especially appreciated the immediate relevance of the research to informing health system design.

The article aims to explore the views of health care consumers and providers on the benefits and challenges surrounding a specialist unit model of care. The research is a qualitative study, drawing on data obtained from workshops and interviews with consumers and providers in a metropolitan area of Australia. The authors identify a number of potential challenges and benefits arising from the specialist unit model of care. The research outlined in the article provides valuable information for policymakers seeking to develop innovative health care models in NSW and elsewhere.

As outlined above the research addresses important issues in designing new models of care delivery. However, from my perspective there are some flaws with the article. I have outlined my major concerns below, as well as identifying a number of minor issues.

MAJOR COMMENTS

1. The authors identify a number of benefits and challenges discussed by consumers and providers. These are described in the abstract, results and conclusion in the manuscript and the authors provide Figure 2 as a summary. However, while there are common threads, the descriptions of the findings in different places do not always seem to align well and it isn't overly clear what the key themes are. Figure 2 could overlap more clearly with the results section – for example, co-morbidities are captured in Figure 2 but the key issue discussed in the results seems to be the potentially narrow scope of care (with co-morbidities being a key risk in that context). The authors note that the challenges and enablers often address the same issues but more could be made of this to guide the overall discussion. Overall, my feeling is that the way the results are presented could be streamlined.
2. Related to the above, greater synthesis would also aid the discussion. The discussion focuses on restating the study findings but could more thoroughly link these back to what this study adds, the implications of the findings and how the findings might influence health system design choices. These issues also extend to the conclusion. In my view, more focus on how the findings relate to the literature and the implications for policy and practice is needed.
3. The methods section provides relatively little detail. I appreciate the authors' note that the methods have been published elsewhere. However, I have not been able to find a copy of the cited report available to read (I have found a reference to it but not the full report, my suggestion is a URL in the citation would be useful.) My main concerns here are, first, that it seems difficult to find more detail and second, it is not clear that the report is a peer-reviewed publication. For these reasons, more detail on the methods and analytical approach would be valuable.
4. Supplementary file 2 appears to be missing from the submitted manuscript. This may be addressed in Suppl file 2, but some description of the Australian health system and how the proposed specialist unit model of care would function (for example, funding arrangements, potential locations relative to populations and services) would help contextualise the study.

MINOR COMMENTS

Line

There are some minor syntaxial and grammatical errors in the manuscript. These should be addressed.

The aims of the study could be clarified. The study is described as aiming to gather consumer and provider views and preferences on specialist units. However, although the views of participants were explored, it isn't clear to me that their preferences are identified or articulated in this study.

61 suggest "managing people with a…" (rather than 'this') specific condition

64 The explanation of specialist units doesn't seem consistent with the above description- for example, children could receive both surgical and medical care.

70 - 74 It isn't clear to me what the benefit of this paragraph is. The evidence of the benefits of specialist units is highlighted in a previous paragraph. This subsequent paragraph gives detail about benefits that are not immediately or obviously relevant to this research.

96 There is some repetition here with line 78

METHODS

I would suggest reviewing the methods section for flow, streamlining and consistency. For example, recruitment methods are described in two different places and it isn't clear that the descriptions of how participants were recruited are consistent. There is also some repetition – for example, it is noted on line 137 and line 157 that a second researcher took detailed notes.

105 The aim of the online questionnaire isn't entirely clear. Was the questionnaire to gather information on participant characteristics, or to ensure eligibility? Were there potential participants who completed the questionnaire but were excluded?

109 Including when the study was conducted would be useful in the study setting

107 Did both consumer and provider participants engage in follow up interviews?

116 How many people responded? If not all eligible respondents were included what was the process for selecting participants?

124 Please reference the standard protocols. Given personal data (including health information) was being collected did the redcap survey include any sort of consent statement?

165 The author AC is not included on the PLOS one generated summary of the research – suggest review that the first author was not accidentally uploaded into the system twice?

199 The section on general comments could be reframed to support the flow of the discussion more clearly – it seems to summarise some key findings before they are addressed in the results.

It isn't always clear to me how the illustrative quotes support the results. For example, the quote in line 360 - it isn't clear how it relates to streamlined pathways and a culture of flexibility. Perhaps more of the quotation would add more context.

	The other quotations in the section are much more clearly aligned. This may just be how I am reading them but I wonder if more context would be useful
420	It may be useful to reflect on other potential strengths and weaknesses of the analytical approach here.
Ethics	Ensure that statements around written and implied consent are consistent in the manuscript
Figure 2	It isn’t clear to me why data on the professional roles of providers are displayed as a bar graph as opposed to a table.
