# Supplementary material for: Consumer and provider perceptions of the specialist unit model of care: A qualitative study
Source: PLoS One. 2024 Feb 12;19(2):e0293025. doi: 10.1371/journal.pone.0293025 (PMC10861032; doi:10.1371/journal.pone.0293025)
Supplement: Attachment — Submitted filename: PONE-D-23-12601 Author response to reviewers.docx [file pone.0293025.s004.docx]

## Author response to Reviewers

## MANUSCRIPT ID: PONE-D-23-12601

## MANUSCRIPT TITLE: Consumer and provider perceptions of the specialist unit model of care: a qualitative study.

All changes to the manuscript have been highlighted.

### **Reviewer 1:**

| REVIEWER COMMENT | AUTHOR RESPONSE | PAGE NUMBER |
| --- | --- | --- |
| Thank you for the opportunity to review this manuscript. I enjoyed reading the article and especially appreciated the immediate relevance of the research to informing health system design.  The article aims to explore the views of health care consumers and providers on the benefits and challenges surrounding a specialist unit model of care. The research is a qualitative study, drawing on data obtained from workshops and interviews with consumers and providers in a metropolitan area of Australia. The authors identify a number of potential challenges and benefits arising from the specialist unit model of care. The research outlined in the article provides valuable information for policymakers seeking to develop innovative health care models in NSW and elsewhere.  As outlined above the research addresses important issues in designing new models of care delivery. However, from my perspective there are some flaws with the article. I have outlined my major concerns in the attached document, as well as identifying a number of minor issues. | We thank the reviewer for their positive endorsement of our paper and introduction. |  |
| The authors identify a number of benefits and challenges discussed by consumers and providers. These are described in the abstract, results and conclusion in the manuscript and  the authors provide Figure 2 as a summary. However, while there are common threads, the descriptions of the findings in different places do not always seem to align well and it isn’t  overly clear what the key themes are. Figure 2 could overlap more clearly with the results section – for example, co-morbidities are captured in Figure 2 but the key issue discussed in the results seems to be the potentially narrow scope of care (with co-morbidities being a key risk in that context). The authors note that the challenges and enablers often address the same issues but more could be made of this to guide the overall discussion. Overall, my feeling is that the way the results are presented could be streamlined. | We thank the reviewer for this point. We have now aligned all the sections to standardise the themes and structure. We have replaced Figure 2 with Table 3 giving a clearer summary of the themes for both consumers and providers. Table 3: Matrix of themes from the workshops for Consumer and Provider participants. \| Barriers, challenges and risks \| \| Enablers and opportunities \|; Theme \| Consumers \| Providers \| Consumers \| Providers; Accessibility \| Not local Poor public transport \| Limited capacity of units \| Local Easy access built in \|; Narrow scope of practice \| Poor fit for some patients with multi-morbidities \| Few patients may meet all the criteria; Unable to deal with co-existing conditions \| \|; Resource and infrastructure concerns \| Understaffing, inexperienced staff \| Less flexible use of beds \| Competent staff and stable funding \|; Patient wellbeing \| Unit may not suit all patients; Alternative models of care may be better \| \| \|; Coordination with other services / departments \| \| May be difficult to appropriately triage and place patients; Need for complementary models of care \| Access to complementary services built in \| Streamlined admission and referral processes; Collaboration across departments; Flexibility in admission criteria; Awareness and expectations \| \| Uncertain if demand would be high or low \| \| Knowledge of the unit’s benefits and limitations would foster appropriate use; Expert care \| \| \| High quality care as providers were experts; One stop shop as allied health co-located \| Well trained, appropriately experienced staff | Pages 12-22  Page 13 |
| Related to the above, greater synthesis would also aid the discussion. The discussion focuses on restating the study findings but could more thoroughly link these back to what this study adds, the implications of the findings and how the findings might influence health system design choices. These issues also extend to the conclusion. In my view, more focus on how the findings relate to the literature and the implications for policy and practice is needed. | Thank you for this point. We have added to the discussion both some extra comments, and as you suggest, a more explicit statement of suggested recommendations.  *Recommendations for specialist units*  *These findings prompt some high level recommendations for the implementation of specialist units that avoid some of the perceived drawbacks. First, specialist units should be determined by a condition’s prevalence in the community and placed in areas of most need. The benefit of a local service can then be realised for more people. Attention to public transport access, parking and, if required family accommodation should be included. Second, funding and infrastructure for the unit needs to be stable to avoid staffing shortages or inappropriate skills mix. Third, robust yet flexible admission and referral processes should be implemented with wide dissemination of benefits and limitations of these units. Fourth, units should be supported and complemented by other models of care such as hospital in the home or integrated care to ensure smooth transitions into and out of the unit.* | p.22-23  P. 24 |
| The methods section provides relatively little detail. I appreciate the authors’ note that the methods have been published elsewhere. However, I have not been able to find a copy of the cited report available to read (I have found a reference to it but not the full report, my suggestion is a URL in the citation would be useful.) My main concerns here are, first, that it seems difficult to find more detail and second, it is not clear that the report is a peer reviewed publication. For these reasons, more detail on the methods and analytical  approach would be valuable. | We have now had our peer-reviewed protocol paper published and it is cited instead of the Report. We have revised the wording to make it clear that methods for this part of the project are given in full.  *Study methods for the entire consultation procedure are described in detail elsewhere ^1^ while specific methods for this part of the study are given in detail below.*  More information on the analysis have also been given:  *Handwritten notes from workshops were de-identified and aggregated into a consumer, and a provider narrative summary (in Microsoft Word) which followed the structure of the question framework. The narrative was then analysed deductively by two senior members of the research team (AC and NR) by collating and synthesising barriers and facilitators, risks, and benefits of the specialist model of care. Verbatim or paraphrased quotes from the handwritten notes were matched to each point. Results were verified by the whole team. Survey responses were analysed using the Microsoft Excel software tool to generate descriptive statistics of the participants’ demographics. These data were compared to the catchment demographics to assess how well they represented the local community.* |  |
| Supplementary file 2 appears to be missing from the submitted manuscript. This may be addressed in Suppl file 2, but some description of the Australian health system and how the proposed specialist unit model of care would function (for example, funding arrangements, potential locations relative to populations and services) would help contextualise the study. | We have checked that Supplementary File 2 has been uploaded.  We have added in a sentence around the Australian context of this study which only considered publicly funded hospitals.  In Australia, specialist units are a key part of the publicly funded health system, strategically incorporated into hospitals and community-based facilities based on a state by state assessment of need, prevalence and access to resources. |  |
| There are some minor syntaxial and grammatical errors in the manuscript. These should be addressed:  The aims of the study could be clarified. The study is described as aiming to gather consumer and provider views and preferences on specialist units. However, although  the views of participants were explored, it isn’t clear to me that their preferences are identified or articulated in this study. | The aim wherever it is stated has been amended to:  *This paper aimed to collate perceptions of local consumers and health providers around specialist care units, as a model of care that may be considered for a new local healthcare facility.* | Pages 2, 6 |
| Line 61 suggest “managing people with a…” (rather than ‘this’) specific condition | This has been changed. | Page 4 |
| Line 64 The explanation of specialist units doesn’t seem consistent with the above description- for example, children could receive both surgical and medical care. | While this is true, children’s hospitals cater to a specialist cohort of patients – no matter what treatments they are receiving – requiring specialist knowledge and skills (e.g., very different medication dosages compared to adults, different vital signs parameters, family-centred care). |  |
| Line 70-74 It isn’t clear to me what the benefit of this paragraph is. The evidence of the benefits of specialist units is highlighted in a previous paragraph. This subsequent paragraph gives detail about benefits that are not immediately or obviously relevant to this research. | We have reviewed these two paragraphs. The reason for the second was that it provided empirical evidence to support the more generic claims in the former paragraph. We have amended to make this clearer.  *The philosophy behind specialist units is that they use interdisciplinary teams to provide expert, best practice for that condition, and these teams are co-located in the one unit…*  *Empirical data supports the effectiveness of this model.* | Page 4 |
| Line 96 There is some repetition here with line 78 | We have reviewed and amended. |  |
| Methods: I would suggest reviewing the methods section for flow, streamlining and consistency. For example, recruitment methods are described in two different places and it isn’t clear that the descriptions of how participants were recruited are  consistent. There is also some repetition – for example, it is noted on line 137 and line 157 that a second researcher took detailed notes. | Thank you for this comment. We have reviewed, restructured and removed repetitive sections. | Pages 6-8 |
| Line 105: The aim of the online questionnaire isn’t entirely clear. Was the questionnaire to gather information on participant characteristics, or to ensure eligibility? Were there  potential participants who completed the questionnaire but were excluded? | Yes, the questionnaire was both an expression of interest and to gather information on participant demographics prior to the focus group (to save time). This has been amended.  *The questionnaire was used to lodge participants’ expression of interest in the study and asked for demographic data including age, gender, location, ethnicity, and contact information…. Everyone who submitted an expression of interest was included.* | Page 6 |
| Line 109: Including when the study was conducted would be useful in the study setting | We state this on page 6:  Recruitment and workshops occurred between July and October of 2021. | Page 6 |
| Line 107: Did both consumer and provider participants engage in follow up interviews? | No. We only had two consumers involved in follow-up interviews. We have added:  Twenty-two consumers from across the health catchment participated in five workshops, *and two of these took part in a follow up interview.* | Page 10 |
| Line 116: How many people responded? If not all eligible respondents were included what was the process for selecting participants? | All people who filled out the questionnaire EOI, were sent information and invited to participate. This has been added    *Everyone who submitted an expression of interest questionnaire and was sent written information about the study. All participants of the workshops gave formally written consent to take part*. | Page 7 |
| Line 124: Please reference the standard protocols. Given personal data (including health information) was being collected did the redcap survey include any sort of consent  statement? | The standard protocol is referring to The *National Statement on Ethical Conduct in Human Research (2023)* that states consent is implied if participants return a survey, i.e., are informed on the landing page of the survey what data will be collected and they proceed and complete it.  In *accordance with the Australian National Statement on Ethical Conduct in Human Research,^2^ clicking on the link and completing the questionnaire was taken as implied consent for their data to be collected. … All data was securely stored on the university’s servers and was used only to report the demographics of participants collectively*. | P.6-7 |
| Line 165: The author AC is not included on the PLOS one generated summary of the research – suggest review that the first author was not accidentally uploaded into the system twice? | Thanks for pointing this out. We will check this when we resubmit. |  |
| Line 199: The section on general comments could be reframed to support the flow of the discussion more clearly – it seems to summarise some key findings before they are  addressed in the results. | We intended this to be a summary of the findings before going deeper into the details. We have renamed the section: Summary of findings | P.12 |
| It isn’t always clear to me how the illustrative quotes support the results. For example, the quote in line 360 - it isn't clear how it relates to streamlined pathways and a culture of flexibility. Perhaps more of the quotation would add more context. The other quotations in the section are much more clearly aligned. This may just be how I am reading them but I wonder if more context would be useful. | We have added some context to this quote and reviewed the others.  *You can’t know everything, even in a specialised unit [so one needs to collaborate with other health professionals].* [Provider 1 Workshop 4] | P.21 |
| Line 420: It may be useful to reflect on other potential strengths and weaknesses of the analytical approach here. | Thanks for this point. We have added in some more limitations.  *Limitations of the work included possible bias of the participants who self-selected and were motivated to have a say in the health facility design. Their views may not necessarily be representative of the rest of the local community. The choice of a dementia specialist unit as the example under discussion may also have limited participants’ responses. The distinctly local and tailored nature of the study means that generalisability should be exercised with caution.* | Page 25 |
| Ethics: Ensure that statements around written and implied consent are consistent in the manuscript. | We have amended the Ethics statement:  *All participants gave informed, written consent to participate in the focus groups and interviews.* |  |
| Figure 2: It isn’t clear to me why data on the professional roles of providers are displayed as a bar graph as opposed to a table. | We prefer to show some descriptive statistics graphically. This makes them more engaging and allows comparisons to be more easily made. |  |

### Reviewer 2:

| REVIEWER COMMENT | AUTHOR RESPONSE | PAGE NUMBER |
| --- | --- | --- |
| This is a clearly written paper that describes outcomes of a broad consultation process, discussing potential models of care for a new health centre. It is encouraging to see this type of work being done in the development phase of new health facilities, and the paper will make an important contribution to the literature. There are two aspects of the paper that I think could be improved prior to publication. | Thank you for your careful reading of the paper and positive response. |  |
| Firstly, the context session providing information about the broader consultation and place of this study within it is clear and helpful. I suggest that it would be best to keep all the information about the broader study here, and then have the rest of the method focus only on the current consultation about specialist care models to avoid any confusion. Hence delete the first sentence under workshops, and instead add a sentence in the context section to state that the consultation process involved a series of workshops (perhaps include the total number of workshops and sample size to give an indication of the overall scope?) with each workshop focussing on a different model of care. Prior to the last sentence of paragraph 1 under context would be a good a place for this (about line 85). | Thank you for this helpful suggestion.  We have amended the context section as suggested and removed this detail from the Methods:  *The consultation involved presenting different, innovative models of health care and eliciting feedback from consumers and health providers separately via two hour workshops.*  The Methods have been restructured and streamlined in response to this and to Reviewer 1’s comments. | Page 5  Pages 6-10 |
| Secondly, the discussion is largely a further presentation of results. It provides the reader with more detail about what the participants said, and summarises the data in different ways, but there is limited integration of the data with what else is known about this field. For example:  - Paragraph 2 talks about “tunnel vision” but includes no references. Are there any studies that have discussed this issue that can be integrated here? The authors state that consumers were making an assumption – is there any data that challenges or supports this assumption from other studies? | We have reviewed the Discussion and rewritten several sections in response to this and Reviewer 1’s comments. We have expanded the section on tunnel vision:  *Greater knowledge and experience with management of a single primary diagnosis intuitively suggests better care and outcomes, and greater confidence from consumers.^3^*  This aligns with a studies that found generalist clinicians with a broad knowledge of how to manage patients with multi-morbidities rather than a single condition were often seen as preferred doctors e.g., ^4^. There was an assumption, particularly among some consumers, that the primary condition that the unit specialised in, resulted in a very narrow and limited scope of practice, which may have quality and safety issues for the patient. While there is little evidence from the literature to support this, Kreindler and colleagues^5^ take a systems level view and state that specialist services can be inflexible and standardised in their processes, suiting only a narrow cohort of patients. | Page 22 |
| - Paragraph 3 talks about integration, and states that “Consumers implied, and providers explicitly stated that this combination of the specialist unit and integrated care was the ideal model”. What do others think about whether this is the ideal model? Does the literature support the view of participants in your study? Sara Kreindler has done some interesting work on this based on a qualitative study involving more than 300 interviews with health services managers in Ontario. She talks about how specialist vs generalist models can be best used, depending on population need (PMID: 33949820). It would be interesting to consider how her findings align (or not) with the perceptions of this group. | Thanks for identifying this paper. We have integrated these authors’ findings in the Discussion.  The perceptions of our consumer and provider participants were considered important to factor into the planning of a new community health facility and were based on their experience of existing services. The design of appropriate services should also be firmly based on an analysis of the community’s health to avoid “population–capacity misalignment”, a situation where the services provided do not meet the community’s needs.^5^ Kreindler and colleagues^5^ call for detailed examination of the health needs of the community before deciding on services: for example, high demand for surgery may be best served by specialist, standardised surgical services, while high demand for services for frail elderly patients with complex care needs would indicate more broad and flexible, generalist services. The current study did not include a detailed examination of local demand for services. However, comments around the desirability of local specialist services and the need for coordination with integrated care models to support flexibility, align with this concept. Both consumers and providers discussed the importance of choosing a specialist service that was of most relevance to the community. | Page 24 |
| - The final paragraphs of the discussion focus on access to care, presenting participants’ perceptions but not references to other literature. There is a lot of literature on access (or lack of access) to specialist care that could be used as a basis for a broader discussion of this issue raised. | This is a good point and it was an issue that was never raised by the consumers. We have added to the Discussion:  Interestingly, consumers did not discuss access to the units themselves, taking for granted that if the patient met the criteria they would be admitted. This is in contrast to an increasing body of literature showing difficulties around access to specialist services leading to unmet needs and poorer clinical outcomes (e.g., ^6,7^). | Page 24 |
| - I note that one issue not discussed in the paper is how one would choose the specialties to be catered for in a specialty model. Did this come up at all in the workshops? If not, was that because the issue was not raised in response to the prompts provided? | We do note that there were comments in both Consumer and Provider Focus groups expressing concern that the specialties chosen might not be relevant to the local community. However, no, the actual choice of specialty areas was not mentioned.  This is covered too in the previous comment above on Page 24. |  |
| It seems to me that specialist are services most likely work well for those they cater for but what are the implications for those who don’t meet the criteria? There are far more potential areas of specialty than could ever be addressed with a limited number of specialty units in a new health service. This is a potentially complex issue that would surely need to be a key consideration if a new health service was to decide to focus on specialty care models. | This is a good point which we didn’t make clear. The type of specialist unit given as the example would be understood as being part of a larger, general hospital. We have noted this:  *Specialist dementia units were understood as being located within a larger public hospital.* | Page 8 |

1.	Carrigan A, Roberts N, Clay-Williams R, et al. Innovative models of care for the health facility of the future: a protocol for a mixed-methods study to elicit consumer and provider views. *BMJ Open.* 2022;12(11):e059330.

2.	National Health and Medical Research Council, Australian Research Council and Universities Australia. National Statement on Ethical Conduct in Human Research In: Canberra: National Health and Medical Research Council; 2023: file:///C:/Users/mq20152886/Downloads/National-Statement-Ethical-Conduct-Human-Research-2023.pdf.

3.	Spencer K, Foster P, Whittamore KH, Goldberg SE, Harwood RH. Delivering dementia care differently—evaluating the differences and similarities between a specialist medical and mental health unit and standard acute care wards: a qualitative study of family carers’ perceptions of quality of care. *BMJ Open.* 2013;3(12):e004198.

4.	Coventry J, Hampton JM, Muddiman E, Bullock A. Medical student and trainee doctor views on the 'good' doctor: Deriving implications for training from a Q-methods study. *Medical Teacher.* 2022;44(9).

5.	Kreindler S, Aboud Z, Hastings S, et al. How Do Health Systems Address Patient Flow When Services Are Misaligned With Population Needs? A Qualitative Study. *Int J Health Policy Manag.* 2022;11(8):1362-1372.

6.	Maistry N, Patel N, Govender T, Grieve A. Voyaging through the paediatric surgical maze: A multi-centre analysis of inter-hospital referrals to Johannesburg, South Africa. *J Pediatr Surg.* 2023;58(2):282-286.

7.	Thompson SG, Barber PA, Gommans JH, et al. Geographic Disparities in Stroke Outcomes and Service Access: A Prospective Observational Study. *Neurology.* 2022;99(4):e414-426.
